# 

**Published:** 1889-03-30

**Authors:** 


PERMANENTLY ENLARGED TO 32 PAGES.

PRICE ONE PENNY.

No. 131, Vol. V.—March 30th, 1889.

[Registered at the General Post Office as a Newspaper.]

Per Annum, 6s. 6d. post free.

Monthly Parts, 6d.; post free, 7½d.

Ditto per Ann. 7s. 6d., post free.

